# Idiopathic mesenteric phlebosclerosis: A case report

**DOI:** 10.1097/MD.0000000000048345

**Published:** 2026-04-24

**Authors:** Jingjing Rao, Yuzheng Xue, Tielong Wu, Yuanyuan Dai, Beilei Xia, Zijun Fan, Haowen Sun, Yingyue Sheng

**Affiliations:** a Wuxi School of Medicine, Jiangnan University, Wuxi, China; b Department of Gastroenterology, Affiliated Hospital of Jiangnan University, Wuxi, China.

**Keywords:** diagnosis and imaging examination, idiopathic mesenteric phlebosclerosis

## Abstract

**Rationale:**

Idiopathic mesenteric phlebosclerosis (IMP) is a rare disease with unclear etiology and pathogenesis. Although most IMP patients have a long-term history of traditional Chinese herbal medicine use, this article reports a case without such a special medical history, which may provide new insights into the disease’s etiology.

**Patient concerns:**

A 59-year-old middle-aged man was admitted to the gastroenterology department due to recurrent upper abdominal pain for 1 month. The fecal occult blood test was weakly positive.

**Diagnoses:**

No significant abnormalities were found in tumor markers, Epstein–Barr virus, cytomegalovirus, T-SPOT, tuberculosis antibodies, or coagulation function. Computed tomography revealed edema and thickening of the intestinal wall in the ascending colon and part of the transverse colon, accompanied by narrowing of the lumen. Multiple calcifications were observed in the right mesenteric vessels, associated with the affected areas. Colonoscopy findings: a circumferential ulcer was noted in the ascending colon at a distance of 75 to 60 cm from the anus, with partial cyanosis of the mucosa and nodular changes. During hospitalization, a series of tests was conducted, and the final diagnosis was IMP.

**Interventions:**

The patient received conservative treatment during hospitalization.

**Outcomes:**

After conservative treatment, the patient’s abdominal pain and hematochezia improved, and the findings of computed tomography and colonoscopy also showed improvement in the condition. To date, the patient has not experienced any significant discomfort during follow-up.

**Lesson:**

Diagnosing IMP requires familiarity with its characteristic features and the ability to interpret relevant imaging findings. It is important to note that long-term use of traditional Chinese herbal medicine is not always the cause. Further research, including the analysis of more clinical cases and the performance of animal experiments, is needed to fully understand the etiology and pathogenesis of IMP.

## 1. Introduction

Idiopathic mesenteric phlebosclerosis (IMP), also known as phlebosclerotic colitis, is a rare type of ischemic colitis. This disease was first reported by Hiramatsu et al in 1991,^[1]^ and Iwashita et al^[2]^ formally named it “Idiopathic mesenteric phlebosclerosis” in 2003. It occurs almost exclusively in Asian populations, with the highest prevalence in Japan and China. The etiology and pathogenesis of IMP remain unclear, but long-term use of herbal medicines has been identified as a potential cause.^[1,3,4]^ Most IMP patients have a history of long-term herbal medicine use. Studies have shown that IMP patients who take Chinese herbal medicine for a long time are more likely to have their ascending colon and cecum affected; therefore, IMP tends to occur in the right half of the colon and may extend to the descending colon, sigmoid colon, or rectum.^[5]^ The prognosis for IMP is generally benign, but surgery may be considered in cases where symptoms persist or intestinal obstruction occurs.^[6]^ Early diagnosis of IMP can be confirmed by computed tomography (CT) and colonoscopy, with treatment options selected based on the severity of the condition, including conservative or surgical management. Characteristic imaging findings include thickening of the colon wall and diffuse calcification of the mesenteric small veins and their intraluminal branches. We report a case of IMP in a patient with no prior history of significant medical conditions.

## 2. Case report

We performed the anonymization of the patient in this study. The patient has provided informed consent for publication of the case. The study was approved by the Medical Ethics Committee of Jiangnan University Affiliated Hospital.

**Figure 1.**
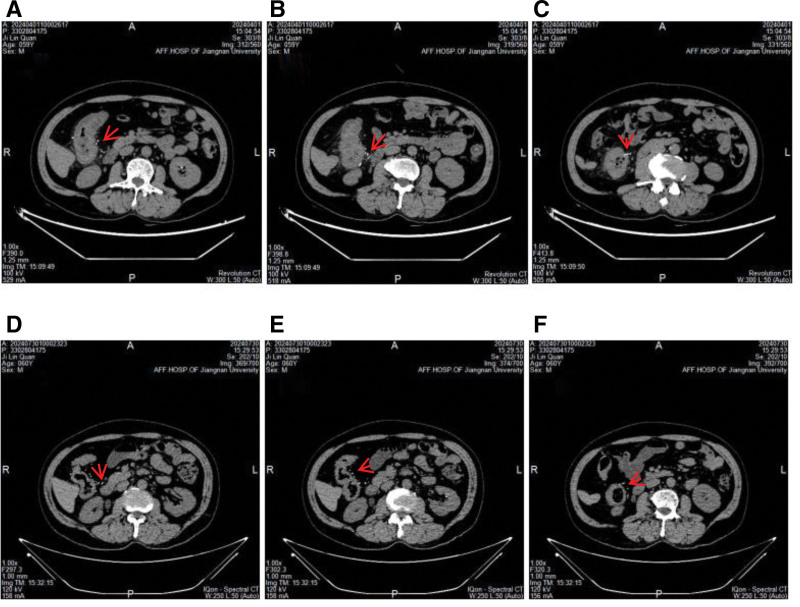
The CT imaging before and after treatment. (A–C) The CT imaging before treatment: calcification of mesenteric vessels, edematous thickening of the bowel wall of the ascending and transverse colon, and a slightly narrowed lumen. (D–F) The CT imaging after treatment: mesenteric vascular calcification as before, significant improvement of bowel wall edema in the ascending and transverse colon, and recovery of luminal narrowing. CT = computed tomography.

**Figure 2.**
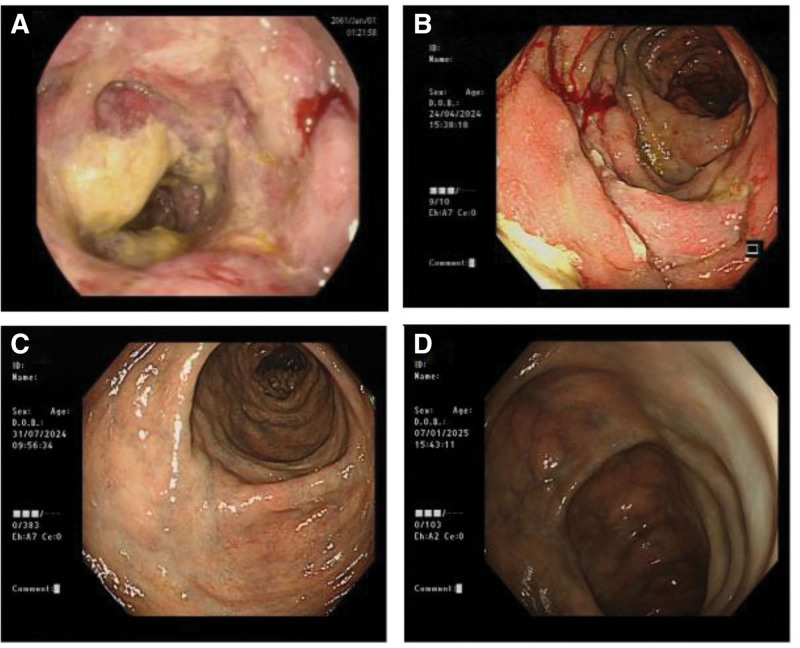
The colonoscopy images before and after treatment. (A) The colonoscopy images before treatment. (B) The colonoscopy images at the initial stage of treatment. (C) The colonoscopy images after treatment. (D) The colonoscopy images during the recent follow-up period.

**Figure 3.**
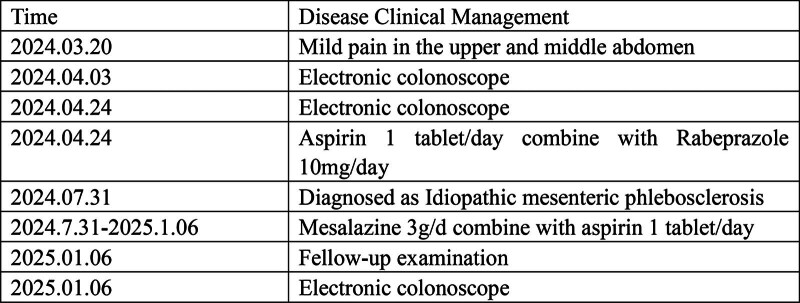
Clinical timeline (initial presentation, investigations, treatments, follow-up). This clinical timeline is divided into 8 time periods, including the specific times of initial presentation, colonoscopy, treatment, diagnosis confirmation, treatment plan adjustment, and follow-up.

The patient is a 59-year-old middle-aged male with no significant past medical history. He presented to the Department of Gastroenterology at Jiangnan University Affiliated Hospital in April 2024 with a 1-month history of recurrent upper abdominal pain. One month prior to the onset of symptoms, the patient suddenly experienced mild discomfort in the upper abdomen without any apparent cause; the pain was intermittent. The patient then underwent a colonoscopy at a community hospital, which revealed a colonic mass, raising suspicion of a colonic tumor. The patient was referred to the oncology department for further evaluation; during this period, a CT scan showed edema and thickening of the wall of the ascending colon and part of the transverse colon, with fluffy density shadows visible around the area, narrowing of the lumen, and small lymph nodes in the abdominal cavity (Fig. 1A–C); as the initial colonoscopy pathology did not indicate malignant lesions, a follow-up colonoscopy was performed, and the report showed a circumferential ulcer in the ascending colon with a crisp texture (Fig. 2A), and a tumor could not be ruled out; a biopsy was taken from the ascending colon, and the pathological examination revealed acute and chronic inflammation of the mucosa with granulation tissue formation. During this period, the patient experienced recurrent abdominal pain with occasional episodes of rectal bleeding, but no evidence of a tumor was found, precluding further diagnostic or therapeutic interventions; only symptomatic treatment was administered. One month later, the patient was transferred to the Department of Gastroenterology at Jiangnan University Affiliated Hospital for further diagnosis and treatment. The patient’s previous colonoscopy and pathology results were reviewed, and the patient was again advised to undergo a colonoscopy with plans to obtain pathology results. The colonoscopy revealed a circumferential ulcer in the ascending colon at 75 to 60 cm from the anus, with partial blue discoloration of the mucosa, nodular changes, and crisp texture; multiple biopsies were taken. Diagnostic considerations: colon mass (malignant tumor? tuberculosis? Lymphoma?) (Fig. 2B); gastroscopy revealed changes consistent with atrophic gastritis. Additional relevant tests were performed, including a complete blood count showing hemoglobin 128 g/L, a fecal occult blood test with a weakly positive result, and no significant abnormalities in tumor markers, Epstein–Barr virus, cytomegalovirus, autoantibodies, immunoglobulins, T-Spot, tuberculosis antibodies, or coagulation function. D-dimer was 0.28 mg/L. Based on the above results, 3 colonoscopies did not reveal any malignant findings; upon reanalysis of the patient’s CT images, swelling and thickening of the intestinal wall with narrowing of the lumen were found in the ascending colon and part of the transverse colon, consistent with the location of the lesions indicated by colonoscopy; in addition, multiple calcifications were found in the right mesenteric vessels, accompanying the lesions. The final diagnosis was “idiopathic mesenteric phlebosclerosis.” During the course of treatment, the patient’s medical history was repeatedly reviewed, and it was confirmed that there was no prior history of oral intake of traditional Chinese herbs or health supplements. Treatment was initiated with oral mesalazine 3 g/d combined with aspirin 1 tablet/d. After 1 week, the patient reported significant improvement in abdominal pain symptoms, and the frequency of bloody stools also decreased markedly. Three months after treatment, a follow-up CT scan showed that the thickening of the ascending colon wall had improved compared with previous findings, with surrounding exudate largely absorbed; multiple calcifications were observed in the mesenteric vessels surrounding the ascending colon (Fig. 1D–F). Follow-up colonoscopy revealed prominent varicose veins in the ascending and transverse colon, with no obvious ulcers observed; scar formation was noted in some areas (Fig. 2C). Discontinued mesalazine, continued aspirin oral administration. Six months later, a follow-up colonoscopy revealed continuous intestinal vascular varicosities in the ascending and transverse colons, with scarring changes in some mucosal areas, maintaining remission (Fig. 2D). To date, the patient has not experienced recurrent abdominal pain or rectal bleeding symptoms. The patient’s treatment process has been compiled into a clear clinical timeline (Fig. 3).

## 3. Discussion

IMP is a rare chronic ischemic bowel disease characterized by intestinal ischemia caused by non-thrombotic, noninflammatory mesenteric fibrosis.^[7]^ Its pathogenesis is unclear, but the ingestion of specific chemicals and toxins that enter the bloodstream may play an important role in the pathophysiological process of IMP. IMP is more prevalent among Asian populations, with the majority of cases originating from East Asian countries such as China, Japan, and South Korea, and only a few cases recorded in Canada, Germany, the United States, and the United Kingdom. This distribution corresponds with the regions where traditional Chinese medicine is consumed. Especially those herbs containing geniposide may be the pathogenesis of the disease. A retrospective study found that 78.7% of patients diagnosed with IMP had used herbs.^[8]^ There are certain differences in the gender distribution of IMP patients in different regions. In Japan, there are more female patients, which may be related to the fact that women are more inclined to use herbal medicine to treat chronic diseases. In China, however, the proportion of male patients is higher, which may be related to the fact that men tend to drink herbal wine.^[9]^ According to literature reviews, IMP is more common in middle-aged and elderly men (median age at onset: 61 years [range: 22–87 years]).^[10]^ The clinical symptoms of IMP are typically nonspecific, making diagnosis challenging. In the early stages of the disease, patients may be asymptomatic. Common manifestations include abdominal pain (70%) and diarrhea (30%), and other concomitant symptoms may include bloody stools, weight loss, and vomiting. If not treated accurately, the severity of the condition may increase.^[3]^ Patients may develop intestinal perforation or intestinal obstruction due to intestinal necrosis or narrowing. Our patient reported multiple episodes of abdominal pain. However, due to the atypical and mild nature of the symptoms, the cause could not be determined. It was only after combining imaging tests that we were able to determine the diagnosis. The imaging characteristics of abdominal CT and colonoscopy are usually the main basis for the final diagnosis and treatment of IMP patients.^[10]^ The most common and characteristic imaging feature is calcification distributed along the mesenteric vein branches and colon wall veins.^[6]^ Although the right colon is more commonly affected, the entire colon may be involved in severe cases.^[6]^ According to a study by Yen et al, the severity of mesenteric vein calcification is typically proportional to the clinical symptoms associated with IMP.^[11]^ Typical endoscopic findings include dark purple or blue intestinal walls in the affected segment of the colon.^[12]^ The pathological features of IMP include mucosal ulcers, ischemic atrophy of the intestinal wall epithelium, and widespread fibrosis and hyalinization of the submucosal layer.^[13]^ The clinical symptoms of IMP are not typical, and so are the laboratory and physical examinations, which makes it easy to be missed or misdiagnosed. IMP needs to be differentiated from the following diseases: ischemic colitis caused by atherosclerosis; lamellar calcification usually occurs in the larger branches of the mesenteric arteries, which is different from the multifocal calcification in IMP. Inflammatory bowel disease primarily comprises 2 subtypes: ulcerative colitis and Crohn disease (CD). Endoscopic findings in ulcerative colitis commonly include friable mucosa, ulcers, erythema, and a loss of the normal vascular pattern. These lesions are typically continuous. In contrast, the inflammation in CD is transmural and patchy, characterized by “skip lesions” where affected segments are interspersed with normal bowel. Characteristic endoscopic findings of CD include aphthous ulcers, serpiginous ulcers, and edematous nodular mucosa that form a “cobblestone” appearance. The mucosal surface may exhibit nodular protrusions and tortuous veins.^[14]^ Colorectal malignancy: imaging reveals mass lesions in the bowel wall, luminal stenosis, and enlarged lymph nodes, with no characteristic calcification. Infectious colitis: CT shows bowel wall thickening, submucosal edema, and inflammatory infiltration, with air-fluid levels visible in some cases, and no characteristic calcification. Our patient presented with symptoms of upper abdominal pain. Abdominal CT showed edema and thickening of the wall of the ascending colon and part of the transverse colon, with fluffy density shadows around it, narrowing of the lumen, and multiple calcification shadows in the adjacent mesenteric vessels. Colonoscopy revealed lesions involving the ascending colon and transverse colon regions. Histological examination showed acute and chronic inflammation of the mucosa with granulation tissue formation. Subsequent communication with the pathology department also revealed thickening and obstruction of some vascular walls. These findings are consistent with the diagnosis of IMP. As there are currently no standardized treatment guidelines or targeted therapies for IMP, the primary treatment strategy for IMP is conservative management. Symptomatic and supportive therapy is recommended, including bowel rest, improving microcirculation, and preventing infection. In addition, aspirin, warfarin, low-molecular-weight heparin, and mesalazine have shown some efficacy in IMP patients.^[9]^ When there is extensive disease (involving the rectum) or severe complications (such as intestinal obstruction, perforation, or massive intestinal bleeding), total or subtotal colectomy is recommended. The presence of intestinal loop dilation, the number of affected colonic segments with venous calcification, and the total score for mesenteric venous calcification on CT have been shown to predict the efficacy of conservative treatment.^[4]^ If treated appropriately, IMP has a good prognosis. In this case, the patient had no history of herbal medicine use, suggesting that we should explore other potential causes, such as the presence of unidentified autoimmune abnormalities that lead to inflammatory and sclerotic changes in the intestines. The mechanisms and etiology of IMP require further investigation through clinical cases and animal experiments.

## 4. Conclusion

The imaging characteristics of CT and/or colonoscopy are crucial for the diagnosis of IMP. Without a thorough understanding of the imaging features of this disease, it is easy to misdiagnose or overlook it in clinical practice. Long-term use of Chinese herbal medicine is not the only causative factor of IMP, and the etiology and pathogenesis of IMP require further investigation through more clinical cases.

## Acknowledgments

The authors wish to acknowledge support from the National Natural Science Foundation of China (Grant Nos. 32372302 and 82405210).

## Author contributions

**Data curation:** Yuzheng Xue, Tielong Wu, Yuanyuan Dai, Beilei Xia, Zijun Fan, Yingyue Sheng.

**Funding acquisition:** Yuzheng Xue, Yingyue Sheng.

**Conceptualization:** Haowen Sun, Yingyue Sheng.

**Supervision:** Yingyue Sheng.

**Writing – original draft:** Jingjing Rao.
